# Role of the S100 protein family in rheumatoid arthritis

**DOI:** 10.1186/s13075-022-02727-8

**Published:** 2022-01-31

**Authors:** Yuan-yuan Wu, Xiao-feng Li, Sha Wu, Xue-ni Niu, Su-qin Yin, Cheng Huang, Jun Li

**Affiliations:** 1 Inflammation and Immune Mediated Diseases Laboratory of Anhui Province, Anhui Institute of Innovative Drugs, School of Pharmacy, Anhui Medical University, Hefei, 230032 China; 2 Postdoctoral Station of Clinical Medicine of Anhui Medical University, Hefei, Anhui China

**Keywords:** S100 protein, Rheumatoid arthritis, Synovial inflammation, Pannus formation, Bone erosion

## Abstract

Rheumatoid arthritis is a chronic systemic autoimmune disease characterized by synovial hyperplasia, inflammatory cell infiltration, and proliferation of inflammatory tissue (angiogranuloma). The destruction of joints and surrounding tissues eventually causes joint deformities and dysfunction or even loss. The S100 protein family is one of the biggest subtribes in the calcium-binding protein family and has more than 20 members. The overexpression of most S100 proteins in rheumatoid arthritis is closely related to its pathogenesis. This paper reviews the relationship between S100 proteins and the occurrence and development of rheumatoid arthritis. It will provide insights into the development of new clinical diagnostic markers and therapeutic targets for rheumatoid arthritis.

## Background

Rheumatoid arthritis (RA) is a chronic systemic autoimmune disease characterized by synovitis. The current global incidence rate is between 0.4% and 1.3% [1], and it is 2–3 times higher in women than in men. Severe RA reduces the life span of patients by 10–15 years. No clinical cure exists, and current drug treatment is only symptomatic and to maintain joint function. Local manifestations of RA in joints include synovial inflammation, exudation, cell proliferation, granuloma formation, destruction of cartilage and bone tissue, and joint tetanism and dysfunction. The extent of changes in joint tissues may differ by site, but the basic pathology remains the same, i.e., (1) diffuse lymphatic or plasma cell infiltration in the limitation tissue and lymphatic follicle formation; (2) vasculitis (with intimal hyperplasia resulting in narrowing of lumen and obstruction) or fibrinoid necrosis; and (3) formation of rheumatoid granuloma [2].

### S100 protein family

At present, there are more than 20 confirmed S100 protein families, which are called S100A1-S100A18, S100B, S100P, S100Z, and S100G [3]. The S100 protein family comprises low molecular weight (9000–14,000 Da) calcium-binding proteins with highly conserved amino acid sequences; they are so named because they dissolve in 100% saturated ammonium sulfate solution [4]. Proteins of the S100 family belong to the EF-hand superfamily, and there are EF-hand domains at the N-terminal and the C-terminal [5]. In cells, most S100 proteins exist in the form of homologous dimers, whereas a few form heterodimers, trimers, and tetramers. They can exist in the form of monomers under special conditions, but dimers exhibit important biological functions.

The S100 protein family, as a Ca^2+^ sensor, regulates many activities inside and outside the cell in a Ca^2+^-dependent manner [6]. By binding with Ca^2+^, conformational changes occur, exposing the binding site to the target protein and resulting in biological effects, such as regulation of gene expression, enzyme activation, cell cycle, cytoskeletal composition, intracellular Ca^2+^ concentration, inflammatory response, and cellular antioxidative processes [7]. Certain members of the S100 family are released outside the cell, regulating the proliferation of target cells, the activity of macrophages, white blood cells, and inflammatory cells produced cytokines or MMPs of synovial fibroblasts.

Their expression is significantly altered in many tumors, neurodegenerative diseases, inflammation, and autoimmune diseases and can serve as a marker for these diseases [8–11]. Table 1 shows the expression of the S100 protein family in RA.

**Table 1:** The expression of S100 protein family in RA

S100	Expression cells	Trend	Function	Receptors	Reference
S100A4	T cells, neutrophils, phagocytes, mast cell chondrocyte.	Serum level ↑	•Induce monocytes to produce proinflammatory cytokines	TLR4, IL-10R, RAGE, CD36	[12–15]
			•Mediates the recruitment and chemotaxis of macrophages		
			•Leads to the increase of MMMP13 and promotes cartilage degradation		
S100A7	Epithelial cells	no data	•The substrate of transglutaminase	RAGE	[10]
S100A8 S100A9	Monocytes, granulocytes, macrophages Osteoclast and fibroblast synovial cells Epithelial cells, endothelial cells	Synovial tissue ↑ Synovial fluid ↑ Serum level ↑	•Promote chemotaxis of macrophages and neutrophils	TLR4, RAGE	[16–18]
			•Endothelial cells, phagocytes, and osteoclasts are activated		
			•Increased cellular inflammation		
			•IL-6 expression of RA FLS is upregulated		
			•Induce Th7 cell differentiation		
S100A11	Ubiquitous expression in various tissues	Synovial fluid ↑ Serum level ↑	•Promote leukocyte chemotaxis and inflammation	RAGE	[19]
S100A12	Granulocytes and monocytes	Serum level ↑	•Recruitment of neutrophils and macrophages	TLR4, RAGE	[20–22]
			•RAGE and TLR4 receptors were activated to induce the expression of IL-1β, IL-6, and TNF-α		
S100B	Chondrocytes, dendritic cells, lymphocytes	No data	•Promote cartilage breakdown	RAGE	[23]

**Fig. 1:**
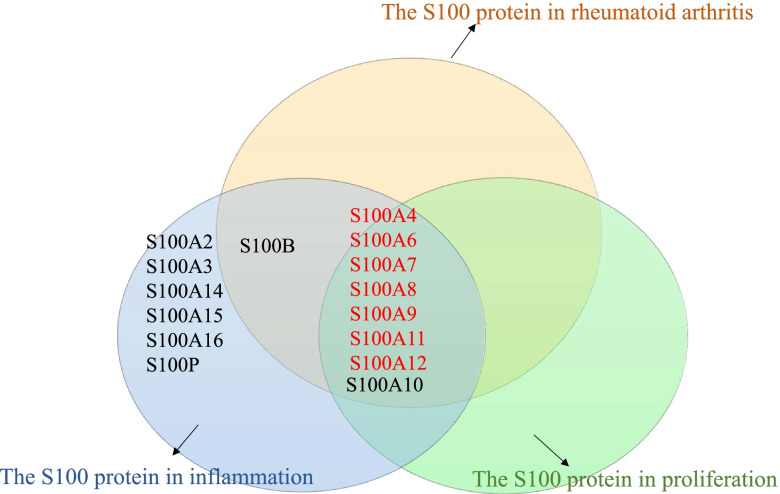
S100 protein was associated with proliferation and inflammation of cells and RA

During the onset of RA, the synovial layer increases from 1 to 3 layers to 10–20 layers, and fibroblast-like synoviocytes (FLS) represent the main cell type in the synovium. FLS can affect macrophage and T cell function by releasing inflammatory factors, chemokines, and metalloproteinases such inflammatory cells, which further aggravates RA [24, 25]. FLS have tumor-like characteristics of uncontrolled proliferation and inflammation [2]. Some S100 protein family members regulate proliferation and inflammation in RA, such as S100A4, S100A6, S100A7, S100A8, S100A9, S100A11, and S100A12 (Fig. 1). Elevated S100 protein concentrations have been detected in the serum and synovial fluid of RA patients, and some S100 proteins can serve as useful biomarkers for monitoring RA activity [26, 27].

### Role of S100 family members in RA

#### S100 proteins and synovial inflammation

**Fig. 2:**
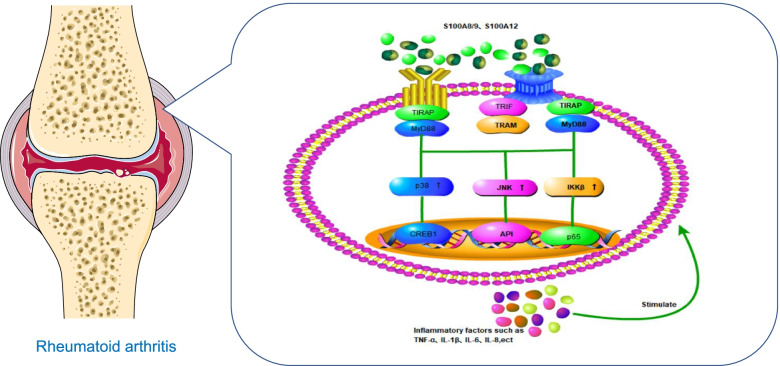
S100A8/9 S100A12 binding of RAGE and TLR-4 receptor S100A8/9S100A12 protein can act as a cytokine to activate TLR or anger-mediated innate immune pathway, TLR, or anger mediated signaling pathway may lead to factors that increase inflammation, such as TNF-α, IL-1β, and IL-6 secretion of inflammatory cytokines, can in turn, stimulate itself, causing a vicious cycle of inflammation

S100 protein members including S100A4, S100A8, s100A9, S100A12, S100A13, and S100A15 regulate inflammation [28, 29]. In RA, S100A4, S100A8, s100A9, S100A11, S100A12, and S100B have been associated with inflammatory responses [30]. S100A8 and S100A9 accounted for 40% and S100A12 accounted for 15% of neutral plasmid proteins [12]. This suggests that neutrophils may be the main source of S100A8/9 and S10012, on the other hand, S100A4 and S100A11 were clearly proved to be produced by a variety of immune cells and by activated synovial fibroblasts. Inflammatory leukocyte infiltration (mainly macrophages) and proinflammatory cytokines play an important role in RA progression [13]. Depending on the type of S100 protein released by neutrophils and monocytes, the cytokine-activated Toll-like receptor (TLR) or receptor for advanced glycation endproducts (RAGE)-mediated innate immune pathways are activated, which increases the levels of tumor necrosis factor (TNF-α), interleukin-1β (IL-1β), and interleukin-6 (IL-6). The increased levels of these inflammatory factors can in turn stimulate neutrophils and macrophages, creating a vicious inflammatory cycle [14, 15]. A schematic representation of the interaction of S100 proteins with RAGE and TLR-4 is shown in Fig. 2.

#### S100A4 and synovial inflammation

S100A4 (also known as metastasin) consists of 101 amino acids with a molecular weight of 12 k Da [28]. S100A4 is the first multifunctional S100 protein identified in metastatic cancer cells, distributed in the cytoplasm, nucleus, and extracellular regions [16]. It not only promotes cancer metastasis but also various other human inflammation-related diseases such as RA [17].

The expression of S100A4 protein in the synovial membrane of the knee joint of RA patients was significantly higher than that in the control group [18], suggesting that S100A4 overexpression positively correlates with the development of RA [28]. The expression of S100A4 mRNA was also found to be increased in the FLS and plasma of RA patients [19]. This is consistent with the involvement of fibroblasts, immune cells, and vascular cells in the production of S100A4 in the synovial tissue [17]. S100A4 levels also increased with an increase in radiation damage and disease progression.

S100A4 recruits the bone marrow cells from inflammatory sites in the body, accelerating the inflammatory process [19, 20]. It was expressed in macrophages, neutrophils, memory T cells, and other lymphocytes and bone marrow cells, verifying the immunomodulatory role of S100A4. It significantly increases the expression of IL-1β, IL-6, and TNF-α by stimulating peripheral blood mononuclear cells, inducing TLR4-mediated inflammatory signaling in mononuclear cells and activating transcription factor (NF)-κ B, extracellular signal-regulated kinase 1/2, and P38 [21]. S100A4 multimers (active form) can also stimulate synovial fibroblasts and regulate apoptosis [22]. These results suggest that S100A4 activates immune and inflammatory responses. Another receptor of S100A4 is the multi-ligand receptor RAGE [21, 23]. After binding to the corresponding ligands, it activates several signaling pathways that affect immunity and wound healing.

#### S100A8 /A9 and synovial inflammation

 S100A8 and S100A9 are only found in vertebrates, and because they are found in myeloid cells, they are also known as migration suppressor associated proteins, and later respectively as bone marrow associated proteins MRP8 and MRP14 [31]. The is S100A8/A9 is also known as calprotectin [32]. S100A8 and S100A9 are usually expressed in the form of heterodimer S100A8/A9 in the early differentiation stage of granulocytes, monocytes, and macrophages and are mainly located in the cytoplasm, cell membrane, and cytoskeleton. Mature macrophages, osteoclasts, keratinocytes, fibroblasts, and microvascular endothelial cells can also induce their expression under certain conditions [33]. S100A8/A9 acts as an inflammatory cytokine and exhibits intracellular and extracellular biological activities. In cells, S100A8/A9 relies on calcium ions to bind to specific cytoskeletal components such as microtubules, vimentin, keratin, and actin filaments. With the participation of calcium, S100A8/A9 forms a tetramer that directly binds to the intracellular microtubules and promotes the rapid polymerization of microtubules; this is the molecular basis for the rapid migration of monocytes and granulocytes during an inflammatory response. Phagocytes release S100A8/A9 by activating protein kinase C and increasing calcium concentration, whereas granulocytes release S1008/A9 when stimulated by lipopolysaccharide and chemokines. The release of extracellular S100A8/A9 plays a biological role in inflammation and immune response as it binds to RAGE receptors and TLR4 [26, 34]. Through synovial fluid analysis of RA and osteoarthritis patients, S100A8, S100A9, and S100A12 were confirmed to be the three most upregulated biomarkers [35]. At the 2016 ACR/ARHP Annual Meeting, Tatsuo Nagai proposed that the expression of S100A9 and S100A12 mRNA in RA BM CD34+ cells was significantly higher than that in OA BM CD34+ cells. In addition, targeted proteomics revealed that S100A8/A9 is a key plasma biomarker for rheumatoid arthritis [27].

Extranodal exudation migration and chemotaxis of neutrophils are the main initiators of local inflammatory pathological changes. S100A8 is a potential chemokine of neutrophils [36]. In vitro, it stimulates the release of L-selectin, regulates the expression and activation of the adhesion molecule MAC-1 (CD11b/CD18), and induces neutrophils to adhere to fibrinogen through MAC-1 [37]. In vivo, S100A8 promotes the migration of neutrophils to the lesion site and participates in the inflammatory response. S100A8/A9 also mediates intracellular inflammatory signal transduction by activating membrane receptors, thereby participating in RA inflammatory response. Extracellular S100A8/A9 mainly binds to two receptors for RAGE and TLR4 subtypes widely present on cell membranes [26]. S100A8 can also stimulate cells to secrete high-mobility Group Box 1, which is also a ligand of RAGE, and along with lipopolysaccharide can synergistically activate the membrane receptor TLR4 and cause inflammatory damage [38]. In addition, S100A8/A9 was found to be involved in the inflammatory response of the scavenger receptor CD36 [39].

#### S100A11 and synovial inflammation

S100A11 (S100C or calgizzarin) is composed of 105 amino acids with a molecular weight of 13 k Da. It is different from other S100 proteins, in that it is mainly expressed in the cytoplasm, whereas other S100 proteins are mainly present in the nucleus [40]. It regulates cell movement, cell cycle, differentiation, transcription, apoptosis, and inflammatory response.

An increase in S100A11 has been reported in the synovial tissue of RA patients, especially in the synovial and inflammatory infiltrates. This finding is consistent with previous studies that have documented the distribution of other S100 proteins associated with RA [41]. The expression of S100A11 in the synovial fluid and serum of RA patients was significantly increased, but there was no significant difference between the upregulation of S100A11 in the serum and that in the synovial membrane of RA patients [41]. In fact, the level of S100A11 in the synovial fluid of cyclic citrullinated peptide-positive patients has been observed to be significantly higher than that in cyclic citrullinated peptide-negative patients [41], which further supports the notion that S100A11 participates in the local inflammatory process of RA.

The level of S100A11 in the synovial fluid is highly correlated with the white blood cell count in the synovial fluid; therefore, the level of S100A11 in the synovial fluid may also be related to the number of immune cells in inflammatory tissues. In addition, recombinant S100A11 promotes the production of IL-6 in the synovial fluid and peripheral blood mononuclear cells, thus enhancing the inflammatory response [42, 43]. Thus, the level of S100A11 in the synovial fluid is significantly correlated with the level of IL-6.

In conclusion, unlike other S100 proteins, S100A11 appears to have weaker cytokine action in RA than other participating proteins, and the systemic level of S100A11 cannot be considered as a biomarker of disease activity. However, S100A11 may enhance the secretion of proinflammatory cytokines in the synovial fluid and form a positive feedback loop in RA [41]. In addition to inflammation, S100A11 may also participate in multiple pathological processes of RA.

#### S100A12 and synovial inflammation

S100A12 protein is composed of 91 amino acids and has a molecular weight of 10.4 k Da. S100A12, like S100A8/A9, is also a type of myeloid protein, which is mainly expressed in neutrophils and mononuclear macrophages under normal conditions, but it can be expressed at high levels in various inflammatory conditions [44].

The healthy synovial tissue without inflammation has no S100A12 [45], suggesting that S100A12 plays a role in the pathogenesis of RA. During the inflammatory process of RA, S100A12 protein is expressed in granulocytes and monocytes, and the expression of S100A12 is closely related to the expression of S100A8 and S100A9. Although its expression level is reduced tenfold compared with that of S100A8/S100A9, and its biological activity is lower than that of S100A8/S100A9, the expression of S100A12 protein is highly correlated with RA progression [46].

The proinflammatory effects of S100A12 in RA include chemotaxis and activation of intracellular signaling cascades that lead to the production of various cytokines and oxidative stress [47]. Primarily, S100A12 produces cytokine-like effects by binding to cell surface receptors including RAGE and TLR-4. It binds to the four extracellular domains of RAGE in the form of a hexamer. The affinity of calcium-bound S100A12 to RAGE is 1000 times higher than that of unbound S100A12. In its hexamer form, S100A12 effectively amplifies intracellular signal transduction, activation of the MAPK kinase and NF-κ B pathways, induction of IL-6, IL-1β, and TNF-α production, induction of the expression of endothelial cell adhesion factors ICAM1 and VCAM1, and mediate the migration and activation of monocytes and the release of cytokine IL-2. Thus, it regulates the activities of lymphoid cells, endothelial cells, and neutrophils and promotes the proinflammatory effects of mononuclear phagocytes [48]. These cytokines can trigger the chemotaxis of neutrophils, monocytes, and lymphocytes and then promote the production of S100A12, thus establishing a positive feedback loop. S100A12 also binds to TLR-4 to similarly activate NF-κB signaling, thereby inducing the expression of various inflammatory molecules and chemokines [49, 50]. S100A12 also has some interaction with human scavenger receptors (including CD36) macrophages, which are implicated in RA pathogenesis [51].

#### Other S100 proteins and synovial inflammation

In addition to the abovementioned proteins, other proteins in the S100 protein family also contribute to synovitis. S100A7 and S100A15, which are directly linked to the innate immune system, are released into the extracellular compartment in response to cell injury, infection, or inflammation and act as proinflammatory signaling proteins [52–54]. In addition, S100B binds to RAGE receptors to promote the release of inflammatory factors, thus participating in the synovial inflammation of RA [55].

#### S100 proteins and pannus formation

Pannus formation occurs in the diseased articular cavity of the vast majority of RA patients. The pannus is mainly composed of new microvessels, proliferating FLS, inflammatory cells, and organized cellulose [56]. Plasma cells, macrophages, and lymphocytes in the pannus have a heightened ability to release immunoglobulins. Pannus formation in the synovium not only cuts off nutrients to the bone but also releases various inflammatory mediators and proteolytic enzymes. Erosion of articular cartilage and subchondral bone, ligaments, and tendon tissue leads to the destruction of articular cartilage, dissolution of subchondral bone, and joint injury (joint dislocation, joint fusion, and ossification).

The S100 protein family is also involved in the pannus formation in RA. Some S100 proteins regulate pannus formation by influencing the release of matrix metalloproteinases (MMPs). As the most important protease for extracellular matrix degradation, MMPs are involved in normal embryonic development and tissue remodeling, as well as inflammatory response, RA, tumor metastasis, atherosclerosis, cerebrovascular disease, and other pathological processes [57]. The abnormal expression of MMPs can promote the development of RA; MMP-2 and MMP-9 are specifically overexpressed in the RA synovial joint. Once MMPs degrade the matrix gel, various proteins including collagen and elastin fibers directly degrade the articular cartilage and through their chemotactic effect recruit various inflammatory cells [58, 59].

Vascular endothelial growth factor (VEGF) is the most important cytokine in promoting angiogenesis in RA [60]. S100A4 is involved in vascular and extracellular matrix remodeling. By activating the mTORC1 signaling pathway, it induces an increase in VEGF level in FLS, directly promoting angiogenesis and accelerating the formation of synovial vascular membranes. Blocking of the endothelial cell migration induced by S100A4 and VEGF binding effectively inhibits angiogenesis [61, 62].

#### S100 protein and bone destruction and erosion

Articular cartilage consists of abundant stroma and a small number of chondrocytes, among which collagen fiber, proteoglycan, and water constitute the cartilage matrix. Chondrocytes synthesize and secrete collagen, proteoglycan, and other interstitial proteins. Cartilage destruction in RA mainly refers to the degradation of the interstitium, which is digested by hydrolyzed proteases. Local bone erosion occurs due to radiological changes in RA that include osteopenia near the joint, focal bone erosion in the subchondral bone, and pannus formation that invades the joint margins. Local bone erosion increases with disease progression and is subsequently a good indicator of the disease severity.

Extracellular S100B is associated with RAGE signaling in chondrocytes, which may be the mechanism underlying osteogenesis in RA [22]. S100A8/A9 induces proteoglycan decomposition, chondrocyte apoptosis. It also upregulates the expression of inducible nitric oxide synthase in macrophages [63]. Nitric oxide is an important inflammatory mediator that induces apoptosis in articular chondrocytes [64]. Therefore, S100A8/A9 may mediate the destruction of RA cartilage.

In addition, extracellular S100A11 activates the p38 MAPK pathway through the RAGE-dependent signaling pathway to accelerate chondrocyte hypertrophy and catabolic metabolism of the matrix [65]. S100A12 is involved in the pathological process of RA joint inflammation and plays an important role in the erosion and damage of joints [66]. It is highly expressed in the synovial fluid and synovial membrane of RA patients but is almost undetectable in the synovial tissue after effective treatment. Its expression may be associated with disease severity, joint rupture, enhanced extracellular matrix proteolysis, autoimmunity, and induction of synovial cell pseudotumor phenotype [67]. Through RAGE signaling, S100A12 promotes the growth of FLS in vitro, resulting in a tumor-like effect, bone erosion, and matrix metalloproteinase production [47].

#### Diagnostic and therapeutic potential of S100 proteins in RA

S100 proteins are considered potential therapeutic targets in the treatment of chronic inflammation. Upregulated S100 protein levels in the serum and synovial fluid are closely related to RA severity and can be used as useful biomarkers. Some S100 proteins are released at the inflammatory site and activate pattern recognition receptors. Since targeted treatment mainly acts at the local inflammatory sites, the risk of systemic adverse reactions is also small.

#### Diagnostic and therapeutic potential of S100A4 in RA

S100A4 levels are increased in proportion to radiographic damage and its further progression in RA patients. High S100A4 levels were associated with a poor clinical response to infliximab and high rate of anti-infliximab antibodies. The finding of a correlation between S100A4 and survivin and Flt3 ligand suggests that these proteins may represent a new cluster of biomarkers predicting radiographic progression and poor treatment response in RA patients [19].

#### Diagnostic and therapeutic potential of S100A8/A9 in RA

Some RA patients who meet clinical remission criteria still have underlying joint inflammation, but the low disease activity makes it difficult to diagnose the condition using conventional clinical laboratory tests. With ultrasound, active synovial inflammation can be detected, but it is not part of the routine clinical laboratory tests. Serum S100A8/A9 protein expression significantly differs between RA patients who only meet the clinical remission criteria but have residual disease activity detected by ultrasound and RA patients who meet the ultrasound remission criteria [35, 68]. This difference in serum S100A8/A9 protein expression may allow us to distinguish RA patients in stable remission from RA patients with residual synovial inflammation. Therefore, we believe that the S100A8/A9 protein level may help clinicians identify patients with active synovitis among RA patients with remission or low activity. Treatment with TNF inhibitors can significantly influence the serum level of S100A8/A9 protein in RA patients [33, 69]. Therefore, monitoring the serum level of S100A8/A9 protein may help evaluate the effect of drug therapy in RA patients. S100A8/A9 level is also a good indicator of the efficacy of methotrexate treatment in RA patients [70]. Therefore, quantification of the changes in serum S100A8/A9 protein level to evaluate the efficacy of anti-rheumatic drugs in early-stage RA patients seems to be a good proposition [32]. Serum S100A8/A9 levels in RA patients were significantly correlated not only with clinical laboratory indicators such as erythrocyte sedimentation rate, C-reactive protein, rheumatoid factor, and disease activity score but also with radiological and clinical assessments of joint injury such as hand X-ray and RA joint injury score [32]. These results suggest that S100A8/A9 is a valuable biomarker for the diagnosis and prognosis of RA.

In addition, the use of S100A9 neutralizing antibody reduces inflammatory response in a mouse model of lipopolysaccharide-synchronized collagen-induced arthritis, and the effect of anti-S100A9 therapy may be a direct result of the inhibition of S100A9-mediated promotion of neutrophil migration and secretion of pro-inflammatory cytokines by monocytes [71]. In conclusion, serum S100A8/A9 protein is a promising biomarker for RA patients with good predictive evaluation of disease activity [70, 72]. It is clear that blocking the expression of this protein may be a new therapeutic strategy for RA.

#### Diagnostic and therapeutic potential of S100A12 in RA

The main aim of RA treatment is to alleviate and eliminate inflammation in joints and prevent joint damage. S100A12 is likely to be a sensitive and specific marker of local inflammation. Moreover, the high level of S100A12 in the local inflammatory site is consistent with its serum level, which allows us to monitor the effectiveness of anti-inflammatory treatment by quantifying the S100A12 serum level [67]. Through RAGE signaling, S100A12 activates the downstream signaling of the NF-κB pathway, which leads to the synthesis of proinflammatory mediators. Therefore, blocking the interaction between RAGE and S100A12 might alleviate RA [67]. Data from a mouse model of arthritis confirmed that S100A12 blocks antibodies to reduce synovial inflammation [73]. Blocking TNF-α in vivo will reduce the expression and release of S100A12 in RA patients and disrupt the feedback loop between TNF-α and S100A12 [51]. The serum concentration of S100A12 decreased after infliximab treatment, suggesting that the treatment inhibits the activation of neutrophils. Almost all standard RA treatments have been shown to affect S100A12 expression, suggesting that S100A12 could be a direct target for successful anti-RA treatment [74]. S100A12 expression level significantly correlates with the levels of other inflammatory markers, as well as clinical evaluation and combined ultrasonic synovitis score [67], indicating that S100A12 is a potential marker of inflammation in RA.

The S100 protein family may be a molecular target for local anti-inflammatory therapy, and inhibition of the expression and release of S100 proteins or blocking the interaction between S100 proteins and innate immune receptors may be effective methods [75]. Blocking cytokines or their downstream signaling by targeting S100 proteins is an effective strategy for RA treatment; however, these treatments often have side effects. Poor patient response and the inability to achieve long-term remission after withdrawal limit the prospect of using these newer strategies [76].

## Conclusion

RA is characterized by aseptic inflammation, which severely and permanently damages various organ systems. S100 proteins can be used not only as inflammatory markers but also as biomarkers in RA. Therefore, further research on the S100 protein family may provide additional evidence for their use in the prevention and treatment of RA. Current research suggests that S100 proteins also influence the treatment response to clinically used drugs; therefore, this protein family has potential significance for the diagnosis, treatment, and prognosis of RA. The focus of future studies should be to determine the specific roles and mechanisms of action of each member of this protein family. It is expected that the findings will allow personalized RA therapy soon.

## Data Availability

We declare that all the data provided above are true.

## Abbreviations

RA: Rheumatoid arthritis
FLS: Fibroblast-like synoviocytes
TLR: Toll-like receptor
RAGE: Receptor for advanced glycation endproducts
TNF: Tumor necrosis factor
IL: Interleukin
MMP: Matrix metalloproteinase
VEGF: Vascular endothelial growth factor

## References

[1] Littlejohn EA, Monrad SU (2018). Early diagnosis and treatment of rheumatoid arthritis. Prim Care.

[2] Smolen JS, Aletaha D, McInnes IB. Rheumatoid arthritis. Lancet. 2016;388(10055):2023-38. 10.1016/S0140-6736(16)30173-8 27156434

[3] Gonzalez LL, Garrie K, Turner MD (2020). Role of S100 proteins in health and disease. Biochim Biophys Acta Mol Cell Res.

[4] Brenner AK, Bruserud Ø (2018). S100 proteins in acute myeloid leukemia. Neoplasia.

[5] Austermann J, Spiekermann C, Roth J (2018). S100 proteins in rheumatic diseases. Nat Rev Rheumatol.

[6] Santamaria-Kisiel L, Rintala-Dempsey AC, Shaw GS (2006). Calcium-dependent and -independent interactions of the S100 protein family. Biochem J.

[7] Donato R (2013). Functions of S100 proteins. Curr Mol Med.

[8] Cristóvão JS, Gomes CM (2019). S100 proteins in Alzheimer's disease. Front Neurosci.

[9] Xiao X (2020). S100 proteins in atherosclerosis. Clin Chim Acta.

[10] Li F, Men X, Zhang W (2014). S100 protein in breast tumor. Indian J Cancer.

[11] Bresnick AR, Weber DJ, Zimmer DB (2015). S100 proteins in cancer. Nat Rev Cancer.

[12] Jung N (2019). Regulation of neutrophil pro-inflammatory functions sheds new light on the pathogenesis of rheumatoid arthritis. Biochem Pharmacol.

[13] Paparo SR (2019). Rheumatoid arthritis and the Th1 chemokine MIG. Clin Ter.

[14] Sims GP (2010). HMGB1 and RAGE in inflammation and cancer. Annu Rev Immunol.

[15] Xia C (2017). S100 proteins as an important regulator of macrophage inflammation. Front Immunol.

[16] Šenolt L (2015). High levels of metastasis-inducing S100A4 protein and treatment outcome in early rheumatoid arthritis: data from the PERAC cohort. Biomarkers.

[17] Nishioku T (2011). Potential role for S100A4 in the disruption of the blood-brain barrier in collagen-induced arthritic mice, an animal model of rheumatoid arthritis. Neuroscience.

[18] Klingelhöfer J (2007). Up-regulation of metastasis-promoting S100A4 (Mts-1) in rheumatoid arthritis: putative involvement in the pathogenesis of rheumatoid arthritis. Arthritis Rheum.

[19] Erlandsson MC (2012). Metastasin S100A4 is increased in proportion to radiographic damage in patients with RA. Rheumatology (Oxford).

[20] Cerezo LA (2014). The metastasis-associated protein S100A4 promotes the inflammatory response of mononuclear cells via the TLR4 signalling pathway in rheumatoid arthritis. Rheumatology (Oxford).

[21] Oslejsková L (2009). Metastasis-inducing S100A4 protein is associated with the disease activity of rheumatoid arthritis. Rheumatology (Oxford).

[22] Uspenskaya YA (2015). Ligands of RAGE-Proteins: role in intercellular communication and pathogenesis of inflammation. Vestn Ross Akad Med Nauk.

[23] Grigorian M, Ambartsumian N, Lukanidin E (2008). Metastasis-inducing S100A4 protein: implication in non-malignant human pathologies. Curr Mol Med.

[24] Bartok B, Firestein GS (2010). Fibroblast-like synoviocytes: key effector cells in rheumatoid arthritis. Immunol Rev.

[25] Li XF (2017). Functional role of PPAR-γ on the proliferation and migration of fibroblast-like synoviocytes in rheumatoid arthritis. Sci Rep.

[26] Wang Q, Chen W, Lin J (2019). The role of calprotectin in rheumatoid arthritis. J Transl Int Med.

[27] Nys G (2019). Targeted proteomics reveals serum amyloid A variants and alarmins S100A8-S100A9 as key plasma biomarkers of rheumatoid arthritis. Talanta.

[28] Ambartsumian N, Klingelhöfer J, Grigorian M (2019). The multifaceted S100A4 protein in cancer and inflammation. Methods Mol Biol.

[29] Shabani F (2018). Calprotectin (S100A8/S100A9): a key protein between inflammation and cancer. Inflamm Res.

[30] Croia C (2019). One year in review 2019: pathogenesis of rheumatoid arthritis. Clin Exp Rheumatol.

[31] Pruenster M (2016). S100A8/A9: from basic science to clinical application. Pharmacol Ther.

[32] Hurnakova J (2015). Serum calprotectin (S100A8/9): an independent predictor of ultrasound synovitis in patients with rheumatoid arthritis. Arthritis Res Ther.

[33] Di Ceglie I (2019). Fc-gamma receptors and S100A8/A9 cause bone erosion during rheumatoid arthritis. Do they act as partners in crime?. Rheumatology (Oxford).

[34] Wang S (2018). S100A8/A9 in inflammation. Front Immunol.

[35] Baillet A (2010). Synovial fluid proteomic fingerprint: S100A8, S100A9 and S100A12 proteins discriminate rheumatoid arthritis from other inflammatory joint diseases. Rheumatology (Oxford).

[36] Jung N, et al. miRNAs Regulate Cytokine Secretion Induced by Phosphorylated S100A8/A9 in Neutrophils. Int J Mol Sci. 2019;20(22):5699. 10.3390/ijms20225699 PMC6887701 31739406

[37] Ahn GO (2010). Inhibition of Mac-1 (CD11b/CD18) enhances tumor response to radiation by reducing myeloid cell recruitment. Proc Natl Acad Sci U S A.

[38] Bouvier D (2020). Study of sRAGE, HMGB1, AGE, and S100A8/A9 concentrations in plasma and in serum-extracted extracellular vesicles of pregnant women with preterm premature rupture of membranes. Front Physiol.

[39] Kerkhoff C (2001). Interaction of S100A8/S100A9-arachidonic acid complexes with the scavenger receptor CD36 may facilitate fatty acid uptake by endothelial cells. Biochemistry.

[40] Eckert RL (2004). S100 proteins in the epidermis. J Invest Dermatol.

[41] Andrés Cerezo L (2017). Calgizzarin (S100A11): a novel inflammatory mediator associated with disease activity of rheumatoid arthritis. Arthritis Res Ther.

[42] Cecil DL (2005). Inflammation-induced chondrocyte hypertrophy is driven by receptor for advanced glycation end products. J Immunol.

[43] Safronova A (2019). Alarmin S100A11 initiates a chemokine response to the human pathogen Toxoplasma gondii. Nat Immunol.

[44] Baillet A (2010). S100A8, S100A9 and S100A12 proteins in rheumatoid arthritis. Rev Med Interne.

[45] Chen YS (2009). Serum levels of soluble receptor for advanced glycation end products and of S100 proteins are associated with inflammatory, autoantibody, and classical risk markers of joint and vascular damage in rheumatoid arthritis. Arthritis Res Ther.

[46] Abdul-Aziez OA (2010). Serum S100A12 and temporomandibular joint magnetic resonance imaging in juvenile idiopathic arthritis Egyptian patients: a case control study. Pak J Biol Sci.

[47] Foell D (2003). Expression of the pro-inflammatory protein S100A12 (EN-RAGE) in rheumatoid and psoriatic arthritis. Rheumatology (Oxford).

[48] Liao H (2004). Use of mass spectrometry to identify protein biomarkers of disease severity in the synovial fluid and serum of patients with rheumatoid arthritis. Arthritis Rheum.

[49] Myles A (2011). Soluble receptor for advanced glycation endproducts is decreased in patients with juvenile idiopathic arthritis (ERA category) and inversely correlates with disease activity and S100A12 levels. J Rheumatol.

[50] Sunahori K (2006). Increased expression of receptor for advanced glycation end products by synovial tissue macrophages in rheumatoid arthritis. Arthritis Rheum.

[51] Nguyen MVC (2019). Prealbumin, platelet factor 4 and S100A12 combination at baseline predicts good response to TNF alpha inhibitors in rheumatoid arthritis. Joint Bone Spine.

[52] Batycka-Baran A (2015). Leukocyte-derived koebnerisin (S100A15) and psoriasin (S100A7) are systemic mediators of inflammation in psoriasis. J Dermatol Sci.

[53] León R (2009). Identification and characterization of binding sites on S100A7, a participant in cancer and inflammation pathways. Biochemistry.

[54] Sun W (2014). Overexpression of S100A7 protects LPS-induced mitochondrial dysfunction and stimulates IL-6 and IL-8 in HaCaT cells. PLoS One.

[55] Karabulut S (2019). Inflammation and neurodegeneration in patients with early-stageand chronic bipolar disorder. Turk Psikiyatri Derg.

[56] Bas DB (2016). Pain in rheumatoid arthritis: models and mechanisms. Pain Manag.

[57] Araki Y, Mimura T. Matrix Metalloproteinase Gene Activation Resulting from Disordred Epigenetic Mechanisms in Rheumatoid Arthritis. Int J Mol Sci. 2017;18(5):905. 10.3390/ijms18050905 PMC5454818 28441353

[58] Burrage PS, Mix KS, Brinckerhoff CE (2006). Matrix metalloproteinases: role in arthritis. Front Biosci.

[59] Itoh Y (2017). Metalloproteinases in rheumatoid arthritis: potential therapeutic targets to improve current therapies. Prog Mol Biol Transl Sci.

[60] Yoo SA, Kwok SK, Kim WU (2008). Proinflammatory role of vascular endothelial growth factor in the pathogenesis of rheumatoid arthritis: prospects for therapeutic intervention. Mediators Inflamm.

[61] Park K (2018). Vascular endothelial growth factor receptor 1 (VEGFR1) tyrosine kinase signaling facilitates granulation tissue formation with recruitment of VEGFR1(+) cells from bone marrow. Anat Sci Int.

[62] Sherbet GV (2006). Metastasis promoter S100A4 is a potential molecular therapeutic target. Cancer Genomics Proteomics.

[63] Hrabar J (2019). Interplay between proinflammatory cytokines, miRNA, and tissue lesions in Anisakis-infected Sprague-Dawley rats. PLoS Negl Trop Dis.

[64] Kao XB (2018). SP600125 blocks the proteolysis of cytoskeletal proteins in apoptosis induced by gas signaling molecule (NO) via decreasing the activation of caspase-3 in rabbit chondrocytes. Eur J Pharmacol.

[65] Amin AR, Islam AB (2014). Genomic analysis and differential expression of HMG and S100A family in human arthritis: upregulated expression of chemokines, IL-8 and nitric oxide by HMGB1. DNA Cell Biol.

[66] Nishida M (2018). S100A12 facilitates osteoclast differentiation from human monocytes. PLoS One.

[67] Nordal HH (2014). The neutrophil protein S100A12 is associated with a comprehensive ultrasonographic synovitis score in a longitudinal study of patients with rheumatoid arthritis treated with adalimumab. BMC Musculoskelet Disord.

[68] de Moel EC (2019). Circulating calprotectin (S100A8/A9) is higher in rheumatoid arthritis patients that relapse within 12 months of tapering anti-rheumatic drugs. Arthritis Res Ther.

[69] Hurnakova J (2017). Relationship between serum calprotectin (S100A8/9) and clinical, laboratory and ultrasound parameters of disease activity in rheumatoid arthritis: a large cohort study. PLoS One.

[70] Obry A (2014). Identification of S100A9 as biomarker of responsiveness to the methotrexate/etanercept combination in rheumatoid arthritis using a proteomic approach. PLoS One.

[71] Cesaro A (2012). An inflammation loop orchestrated by S100A9 and calprotectin is critical for development of arthritis. PLoS One.

[72] Kang KY, Woo JW, Park SH (2014). S100A8/A9 as a biomarker for synovial inflammation and joint damage in patients with rheumatoid arthritis. Korean J Intern Med.

[73] Nordal HH (2016). Calprotectin (S100A8/A9) and S100A12 are associated with measures of disease activity in a longitudinal study of patients with rheumatoid arthritis treated with infliximab. Scand J Rheumatol.

[74] Wittkowski H (2007). Effects of intra-articular corticosteroids and anti-TNF therapy on neutrophil activation in rheumatoid arthritis. Ann Rheum Dis.

[75] Chen SJ, et al. Immunopathogenic mechanisms and novel immune-modulated therapies in rheumatoid arthritis. Int J Mol Sci. 2019;20(6):1332. 10.3390/ijms20061332 PMC6470801 30884802

[76] Conigliaro P (2019). Challenges in the treatment of rheumatoid arthritis. Autoimmun Rev.

